# Improving Health-Promoting Effects of Food-Derived Bioactive Peptides through Rational Design and Oral Delivery Strategies

**DOI:** 10.3390/nu11102545

**Published:** 2019-10-22

**Authors:** Paloma Manzanares, Mónica Gandía, Sandra Garrigues, Jose F. Marcos

**Affiliations:** Department of Biotechnology, Instituto de Agroquímica y Tecnología de Alimentos (IATA), Consejo Superior de Investigaciones Científicas (CSIC), 46980 Paterna, Valencia, Spain; mgandia@iata.csic.es (M.G.); sgarrigues@iata.csic.es (S.G.); jmarcos@iata.csic.es (J.F.M.)

**Keywords:** nutraceuticals, functional foods, food-derived bioactive peptides, hypertension, type 2 diabetes, oxidative stress, structural requirements, computational methods, rationally designed peptides, oral delivery strategies

## Abstract

Over the last few decades, scientific interest in food-derived bioactive peptides has grown as an alternative to pharmacological treatments in the control of lifestyle-associated diseases, which represent a serious health problem worldwide. Interest has been directed towards the control of hypertension, the management of type 2 diabetes and oxidative stress. Many food-derived antihypertensive peptides act primarily by inhibiting angiotensin I-converting enzyme (ACE), and to a lesser extent, renin enzyme activities. Antidiabetic peptides mainly inhibit dipeptidyl peptidase-IV (DPP-IV) activity, whereas antioxidant peptides act through inactivation of reactive oxygen species, free radicals scavenging, chelation of pro-oxidative transition metals and promoting the activities of intracellular antioxidant enzymes. However, food-derived bioactive peptides have intrinsic weaknesses, including poor chemical and physical stability and a short circulating plasma half-life that must be addressed for their application as nutraceuticals or in functional foods. This review summarizes the application of common pharmaceutical approaches such as rational design and oral delivery strategies to improve the health-promoting effects of food-derived bioactive peptides. We review the structural requirements of antihypertensive, antidiabetic and antioxidant peptides established by integrated computational methods and provide relevant examples of effective oral delivery systems to enhance solubility, stability and permeability of bioactive peptides.

## 1. Introduction

Nutraceuticals are defined as isolated food-derived bioactive molecules, which provide physiological benefits beyond basic nutrition [1]. Among them, bioactive peptides are associated with numerous health benefits and their potential therapeutic use has been extensively discussed during the last decades [2,3,4]. Food-derived bioactive peptides are embedded within the primary structure of dietary proteins and, once released by food processing or digestive enzymes during gastrointestinal transit, exert beneficial effects upon human health. Bioactive peptides range in size from 2 to 50 amino acid residues and, based on their inherent amino acid composition and sequence, exhibit different activities affecting the digestive, cardiovascular, immune and nervous systems [5].

The growing prevalence of lifestyle-associated diseases, including obesity, type 2 diabetes and cardiovascular disease, and the need to reduce risks of occurrence of these diseases, have led scientists and industry to focus on the biological properties of food-derived peptides [6]. Moreover, some of these peptides are multifunctional, which is very attractive for dietary approaches and for functional food development [3,7].

In particular, interest has been directed towards the effects of bioactive peptides on hypertension, one of the main factors for cardiovascular diseases, which represent a major cause of mortality in developed countries. The renin–angiotensin system (RAS) is the most recognized humoral system for the control of blood pressure, and its dysfunctions are involved in the pathophysiology of hypertension. Briefly, prorenin is converted to active renin by a trypsin-like enzyme. Renin cleaves angiotensinogen to form angiotensin I. Angiotensin I-converting enzyme (ACE) hydrolyses both the inactive angiotensin I into vasoconstrictor angiotensin II and the vasodilator bradykinin into an inactive metabolite leading to blood pressure up-regulation [8]. Several current antihypertensive drugs target on the RAS at different points of the cascade: ACE, upstream renin activity, or downstream angiotensin receptors [9]. ACE inhibition is the goal of many food-derived antihypertensive peptides [10,11], although renin is also the target for some bioactive peptides [12].

Another important field for the application of food-derived bioactive peptides is the management of type 2 diabetes, which affects an increasing number of people worldwide. Therapeutic approaches are aimed at decreasing postprandial hyperglycemia mainly by the inhibition of dipeptidyl peptidase-IV (DPP-IV), which degrades and inactivates the incretin hormones glucagon-like peptide 1 and glucose-dependent insulinotropic polypeptide. These incretins induce an increase in insulin secretion during the postprandial phase as a response to food intake and, therefore, inhibition of the DPP-IV activity controls hyperglycemia in type 2 diabetes [13]. Another therapeutic approach in the management of postprandial hyperglycemia is based on the inhibition of digestive enzymes involved in carbohydrate metabolism, such as α-amylase and α-glucosidase. The former hydrolyses starch and complex carbohydrates into oligosaccharides, which are further hydrolyzed by intestinal α-glucosidase to release free glucose. Inhibition of both enzymes has been suggested as an effective strategy for decreasing polysaccharide substrate availability for glucose release in the gut [14]. DPP-IV inhibition is the most aimed target for food-derived antidiabetic peptides [15,16].

**Figure 1:**
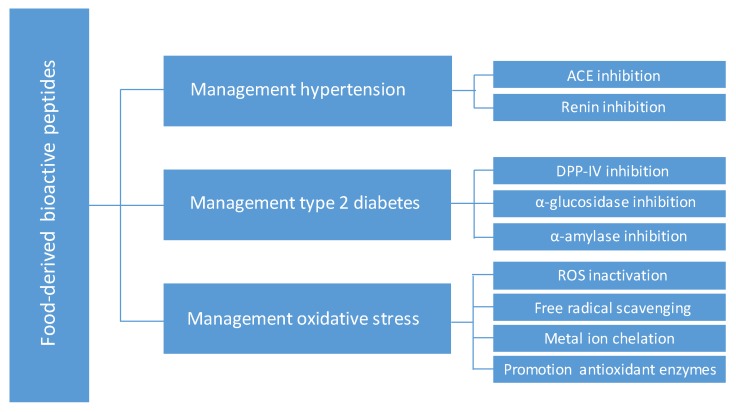
Main targets of food-derived bioactive peptides in the management of hypertension, type 2 diabetes and oxidative stress.

Oxidative stress results from the metabolic reactions that use oxygen and represents a disturbance in the equilibrium status of prooxidant/antioxidant reactions in living organisms. Excess of reactive oxygen species (ROS) can damage cell structures, including lipids and membranes, proteins, and DNA. Oxidative stress is strongly correlated with cardiovascular and neurodegenerative diseases, diabetes and inflammatory disorders [17]. So far, hundreds of antioxidant peptides have been identified from various dietary proteins, and extensive research has explored their ability to protect cells against oxidative stress through inactivation of ROS, free radicals scavenging, chelation of pro-oxidative transition metals and promoting the activities of intracellular antioxidant enzymes [18,19,20]. Figure 1 shows the main targets of food-derived bioactive peptides in the management of hypertension, type 2 diabetes and oxidative stress.

**Figure 2:**
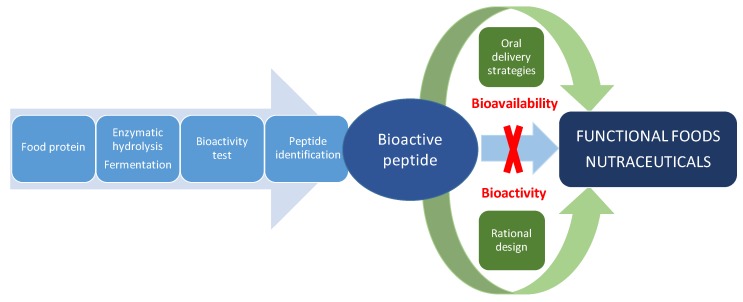
Pharmaceutical approaches to solve the main challenges of food-derived bioactive peptides for the development of functional foods and nutraceuticals.

Despite the tremendous potential of food-derived bioactive peptides, they have intrinsic weaknesses, including poor chemical and physical stability, and a short circulating plasma half-life, which must be considered for their application as nutraceuticals or in functional foods. Some of these disadvantages have been addressed for therapeutic peptides in drug development or antimicrobial peptides for plant protection by the rational design of novel non-natural peptides with improved biological activity, specificity or bioavailability [21,22]. In addition, strategies for effectively delivering therapeutic peptides by the oral route have also contributed to protect peptides from pre-systemic degradation and improve epithelial permeation [23,24]. Undoubtedly, food-derived bioactive peptides research can benefit from pharmaceutical approaches for the discovery, design and development of nutraceutical peptides with improved health-promoting effects as summarized in Figure 2. This review assesses the utilization of rational design and oral delivery strategies for the improvement of bioactive peptides derived from dietary proteins. First, we review the structural requirements of antihypertensive, antidiabetic and antioxidant peptides on which the rational design is based. Finally, we discussed the progress made in improving solubility, stability and permeability of food-derived bioactive peptides by means of oral delivery strategies.

## 2. Structural Requirements of Bioactive Peptides

Integrated computational methods including quantitative structure–activity relationships (QSAR) models, molecular docking and molecular dynamics simulations, have been widely used in drug development and have also found application in food-derived bioactive peptide research [25]. These technologies allow the association of the structural characteristics of peptides to their biological properties, the prediction of bioactivity and physicochemical properties of peptides, and illustrate molecular interaction mechanisms. Attempts to understand the structural requirements of antihypertensive, antidiabetic and antioxidant peptides are summarized below and relevant examples of rationally designed sequences are provided.

### 2.1. Antihypertensive Peptides

**Figure 3:**
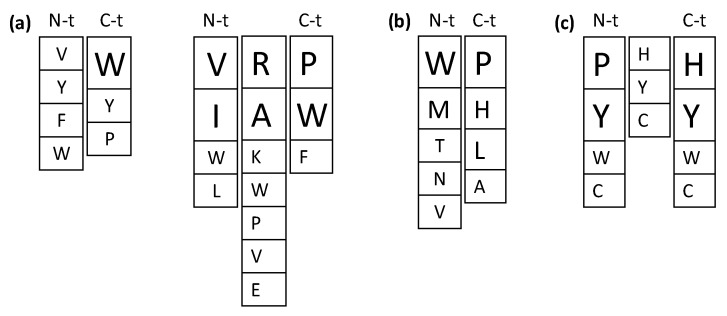
Preferred amino acid residues within the sequence of angiotensin I-converting enzyme (ACE) inhibitory di- and tripeptides (**a**), dipeptidyl peptidase-IV (DPP-IV) inhibitory dipeptides (**b**) and antioxidant tripeptides (**c**). Font size indicates the most favorable residues for each position.

Despite the numerous studies on the ACE inhibitory activities of food-derived bioactive peptides, the relationship between their structure and the ACE inhibitory activity has not yet been fully established, indicating the complexity and/or multi-target nature of the inhibitory mechanism. Moreover, strong evidence points to other in vivo antihypertensive mechanisms beyond ACE inhibition [7,12,26], which hinders the design and development of improved antihypertensive sequences. Figure 3a summarizes preferred amino acid residues within the sequence of ACE inhibitory di- and tripeptides based on studies discussed below. Table 1 shows predicted and novel antihypertensive sequences obtained by computational methods and rational design based mainly on ACE inhibition, although few examples of renin inhibitory sequences are also provided.

The pioneering studies about snake venom ACE inhibitory peptides indicated that their C-terminal tripeptide residues play a predominant role in competitive binding to the active site of ACE, with the optimal sequences being WAP and FAP [27,28]. These structure–activity studies allowed the design of clinically important ACE inhibitors that are analogues structurally related to the dipeptide and tripeptide sequences AP and FAP. Among them, captopril (D-3-mercapto-2-methylpropanoyl-L-proline) was the first orally active specific ACE inhibitor [29]. Later on, ACE competitive inhibition studies using dipeptides revealed residues V, I, A, R, Y, and F, and residues W, Y, F, P, I, A, L, and M as preferred in penultimate and ultimate positions, respectively [30]. Since the difference between the inhibitory potency of the most potent dipeptide tested (VW) and the weakest inhibitor (PG) was 10,000-fold, authors stated that ACE is highly specific to the terminal dipeptide residues of peptide substrates or competitive peptidic inhibitors [30]. Regarding food-derived ACE inhibitory peptides, it has been established that they share certain structural features: they are short in length, and their potency is strongly influenced by their C-terminal tripeptide sequence, which usually contains hydrophobic amino acids but also P, K, or R residues [11]. Relevant examples of such peptides are the well-known milk tripeptides VPP and IPP, the first peptides for which blood pressure lowering properties were reported [6,31,32,33,34].

QSAR modeling of food-derived ACE inhibitory peptides complements the experimental work. Based on an ACE inhibitory peptide database including 168 dipeptides and 140 tripeptides from literature, Wu and coworkers [35] applied a QSAR model to establish the structural requirements of ACE inhibitory di- and tripeptides. For dipeptides, amino acid residues with bulky side chains as well as hydrophobic side chains such as F, W and Y were preferred. For tripeptides, the most favorable residues for the C-terminus were aromatic amino acids, while positively charged amino acids were preferred for the middle position, and hydrophobic amino acids were preferred for the N-terminus. These requirements were then used to predict three dipeptides (FW, WW and YW) and four tripeptides (VRF, IKP, LRW and LRF) located within the primary structure of food proteins [35]. Remarkably, designed sequences LRW, IKP and FW were experimentally validated, and showed an inhibitory potency higher than that of VPP and IPP. Recently, a data set that contains 141 ACE inhibitory dipeptides was used in a single QSAR model with 16 amino acid descriptors [36]. This complex model allowed the prediction of five novel ACE inhibitory dipeptides (CW, TW, HW, QW and CY) and showed that the most correlated properties to ACE inhibitory activity were hydrophobicity, steric, and electronic properties, highlighting the greatest contribution of C-terminal amino acid [36]. This and the previous QSAR studies point to the importance of W, and in general aromatic residues, for the ACE inhibitory activity of peptides.

The potential for molecular docking simulation to predict ACE inhibitory sequences has been shown in different studies. In this context, docking of all potential dipeptides to the ACE active site identified the novel dipeptides DW and WP, which are contained in the primary sequence of several food proteins and predicted to be stable in the intestine, and which were also confirmed by in vitro experiments [37]. It must be stressed, however, that not all the dipeptides predicted by docking in this study were potent ACE inhibitors, pointing to the importance of experimental confirmation. Additionally, molecular docking simulations of ACE inhibitory milk di- and tripeptides allowed the evaluation of structural features and, in agreement with QSAR studies, revealed that the highest ACE inhibitory dipeptides had W at their C-terminus (KW and VW) [38]. In the docking simulation of such dipeptides to ACE, the W residue is located within the hydrophobic core of the ACE active site. Among tripeptides, the highest ACE inhibitory activity corresponded with those having P (MKP, FAP, VAP, IPP, VPP and LRP) or Y (IVY) at the C-terminus. Similarly to the W residue in dipeptides, both residues docked deeply into the hydrophobic core of ACE. This study also highlighted the importance of hydrophobic residues at positions 2 and 3 for ACE inhibitory activity [37]. Another approach used a computational protocol based on molecular docking applied to a tripeptide library to select five W-containing sequences (WCW, IWW, WWW, WWI and WLW) as potent in vitro ACE inhibitors [39].

Involvement of W in the in vitro ACE inhibitory activity of peptides seems clear from QSAR and docking studies. In particular, the relevance of W at the C-terminal end of peptides was proven as a successful rationale for the design of novel tripeptides with improved bioactivity [40]. Novel rationally designed tripeptides of sequences VKW, YAW, KYW and TAW, derived from the dipeptides VK, YA, KY and the tripeptide TAY identified from an oyster hydrolysate [49], increased their potency 27–1450 times compared to their corresponding parental sequences [40].

The relevance of W in the sequence of renin inhibitors has also been established. In particular, QSAR modeling has been used for studying the structure activity relationship of a group of natural renin-inhibiting dipeptides identified in an enzymatic pea protein hydrolysate [47]. The most potent predicted peptides (IW, LW, VW and AW) all possessed W at the C-terminus, and branched-chain aliphatic residues, L or I, at the N-terminus. The authors suggested IW as template for the development of highly active low molecular size antihypertensive peptides and peptidomimetics. Interestingly, all the predicted dipeptides had been previously characterized as ACE inhibitors and were shown to lower blood pressure in spontaneously hypertensive rats (SHRs) [50,51,52]. Further characterization of W-containing peptides showed that are readily absorbed in the gastrointestinal tract after oral administration and inhibit plasma human ACE activity [53]. Moreover, peptides exerted strong ACE-inhibiting effects in human umbilical vein endothelial cells (HUVECs) and aorta, and effectively counteracted angiotensin-induced vasoconstriction and preserved endothelium-dependent vessel relaxation [54]. Whether these W-containing peptides act as dual in vivo inhibitors of renin and ACE deserves further research.

In this regard, the strategy of searching food-derived peptides capable of simultaneously inhibiting more than one target (receptor or enzymatic activity) involved in the hypertension pathophysiology is of utmost interest. The feasibility of peptides to act as dual inhibitors of renin and ACE has been explored by a computational protocol that combined QSAR modeling, molecular dynamics simulation and analysis of peptide binding free energy to renin or ACE [48]. Authors concluded that it was possible to generate three promising candidate sequences such as RYLP, YTAWVP and YRAWVL with good inhibitory potency against both enzymes. However, it was stated that for short peptides such as dipeptides, it is hard to link the renin and ACE inhibitory activities and that longer peptides such as tetrapeptides and pentapeptides are better candidates for exerting dual inhibition. Limited structural variation of dipeptides also hampered the design of potent ACE inhibitory dipeptides without bitter tastes [55]. By contrast, it seems that the structural diversity of tripeptides is sufficient to design multifunctional tripeptides with strong ACE inhibitory potency, high antioxidant activity and weak bitter taste [56].

P residues are also common and relevant in food-derived antihypertensive peptides, as discussed earlier. The presence of P at the C-terminus improved the affinity of peptides for ACE [30] and could provide resistance to digestion in the gastrointestinal tract [57]. In fact, this is the case of the deeply characterized antihypertensive lactotripeptides VPP and IPP. Approaches such as QSAR modeling and docking simulation have also identified bioactive sequences with a P residue at the C-terminus. Tripeptides IVP, INP, IQP and VIP, which were derived from the primary sequence of cow milk proteins, were predicted by a QSAR model based on cow milk- and human milk-derived antihypertensive tripeptides [41]. Moreover, authors stated that the tripeptide IVP was as effective as IPP in SHRs in the conditions tested. As already discussed in this review, docking simulation of milk-derived ACE-inhibitory tripeptides also highlighted the importance of C-terminal P and the design of tripeptides MKP, FAP, VAP and LRP [38].

However, the role of a specific C-terminal residue in enhancing ACE inhibition in peptides longer than tripeptides is more complex. In fact, the relationship between the structure and ACE inhibitory activity for longer peptides has not yet been fully established. QSAR modeling of milk-derived ACE inhibitory peptides up to a length of six amino acids found a relationship between the composition of the two most external amino acids at the C-terminal region and the inhibitory potency, but no relationship was apparent for the two amino acids at the N-terminal region [58]. Moreover, the correlation between C-terminal amino acids and activity decreased in longer peptides, suggesting the influence of steric effects [58]. It has also been proposed that the C-terminal tetrapeptide [59] or pentapeptide [60] determine the structural requirement for efficacious inhibition of ACE activity. Regarding the role of a C-terminal P residue in longer peptides, it is worthwhile to mention the approach of Ding and coworkers to improve the antihypertensive activity of VPP and IPP [42]. With this aim, the hexapeptides VPPIPP and IPPVPP were designed and their in vitro ACE inhibitory activity and in vivo antihypertensive effect were evaluated. Regarding in vivo effects, VPPIPP caused a more potent and faster effect than that caused by IPP, in contrast to that observed in vitro. However, both peptides only caused a transient blood pressure lowering effect since the systolic blood pressure (SBP) recovered to the initial state 5 h after oral administration [42]. Similarly, sequences based on N-terminal elongations of the tripeptide LRP such as KLRP, YKLRP, PYKLRP or DPYKLRP induced significant reductions in SBP in in vivo assays of antihypertensive effect, although only the effect of sequences DPYKLRP and LRP remained significant up to 24-h post-administration [61]. Based on in vivo effects and in vitro gastrointestinal digestions, authors suggested that the sequence LRP is a digestion fragment that could contribute to the blood pressure lowering effects of parental peptides [61]. Whether tripeptides with P at the C-terminus such as VPP, IPP and LRP are feasible lead sequences for the design of longer peptides with improved antihypertensive effect deserves future research.

Many food-derived antihypertensive peptides share a C-terminal F residue [10] fulfilling the rule proposed by Cheung and coworkers [30]. Based on this observation, a database of 53 sequences was recently constructed and evaluated by three-dimensional (3D)-QSAR, molecular docking and bioactivity. This study allowed the identification of four novel potent in vitro ACE-inhibitory tripeptides of sequences GEF, VEF, VRF and VKF [43]. Moreover, the established models suggested that the crucial factor affecting the ACE inhibitory activity of GEF and VKF was the molecule volume, while for VEF and VRF it was the sequence hydrophobicity. Although the models predicted the in vitro ACE inhibitory activity of tripeptides with a C-terminal F residue, unfortunately no data about in vivo antihypertensive effect were provided.

The presence of the C-terminal FW motif described in ACE inhibitory peptides [30,35] led to the evaluation of the bioactivity of a set of lactoferricin B (LfcinB)-related peptides sharing this C-terminal sequence [44]. In vitro and ex vivo functional assays allowed the selection of two hexapeptides, PACEI32L (RKWHFW) and PACEI34L (RKWLFW) with inhibitory effects on both ACE activity and ACE-dependent vasoconstriction [44]. The study concluded that residues at the third C-terminal position influence the in vitro ACE inhibitory effect since C-terminal tripeptides HFW and LFW are preferred over RFW, whereas P and W at the third C-terminal position do have equal effects. Moreover, this study also suggested the importance of the fourth C-terminal position and confirmed the complex structure–activity relationship in peptides longer than three residues. Interestingly, the two most potent sequence-related hexapeptides PACEI32L and PACEI34L were used as lead sequences for the design and screening of a second generation of heptapeptides with improved ACE inhibitory effects [45]. The rationale for the design of heptapeptides was the combination of residues F, H and L in the C-terminal positions because they are preceding the C-terminal W residue in the lead hexapeptides and also because they are the three last C-terminal residues of angiotensin I (DRVYIHPFHL), the natural substrate of ACE. Remarkably, the designed heptapeptides showed improved ACE inhibitory properties in vitro, ex vivo and in vivo, when compared to the parental hexapeptides. Among them, one selected heptapeptide (PACEI50L; RKWHFLW) induced antihypertensive effects in SHRs after oral and intravenous administration and appeared to be safe in cytotoxicity assays [45]. The change in the configuration of the penultimate amino acid residue (H) from C-terminal of angiotensin I is also a successful strategy recently described for drug design. The custom-designed tripeptide FhL (where lowercase letter indicates D-amino acid) strongly interacts with the critical active site amino acid residues of ACE, decreases blood pressure and the levels of circulating angiotensin II [62].

The milk protein lactoferrin (LF) and its proteolytic fragment lactoferricin (Lfcin) are a promising source of ACE inhibitory and antihypertensive peptides whose characterization has provided information about determinant residues in the relationships between sequence and inhibitory potency [7]. Characterization of a set of sequence-related LfcinB-derived peptides suggested a positive relationship between N-terminal sequence and inhibitory activity, since the sequences LfcinB_19–25_ (CRRWQWR), LfcinB_18–25_ (KCRRWQWR) and LfcinB_17–25_ (FKCRRWQWR), derived from elongations at the N-terminal end of LfcinB_20–25_ (RRWQWR), showed higher in vitro potency than the parental sequence [63]. The presence of the positively charged R residue at the C-terminus of the above LfcinB-derived peptides reinforced the importance of the R residue at the C-terminal position described for milk-derived peptides [64]. Moreover, differences between Lfcin_17–31_ (FKCRRWQWRMKKLGA) and Lfcin_17–32_ (FKCRRWQWRMKKLGAP) supported the role of a C-terminal P residue in enhancing ACE inhibitory activity of much longer peptides, as already described for short peptides [30]. With the aim of studying the relationship between activity and the last three amino acid residues of the β-casein-derived peptide LHLPLP, which exhibits potent antihypertensive activity, several modified peptides with substitutions of amino acids in the antepenultimate, penultimate and ultimate positions were synthesized and their ACE-inhibitory activity was measured [46]. The evaluation of seven synthetic peptides (LHLPLL, LHLPLR, LHLPAP, LHLPYP, LHLPGP, LHLYLP and LHLWLP) showed that substitution of L in the penultimate position by G (LHLPGP) and the substitution of P in the C-terminal position by R (LHLPLR) improve the in vitro ACE inhibitory activity.

Another significant example of sequence-activity relationship inferred from the characterization of sequence-related peptides is that of the rice dipeptide RF, which exhibited potent vasorelaxing activity and decreased blood pressure in SHRs [65]. The antihypertensive activity of the elongated peptide IHRF was more potent and long lasting after oral administration than that of the parental sequence. Given that peptides IH and IHR were inactive while HRF was active, authors suggested that RF at C-terminal is critical for the activity and that elongations at N-terminal are tolerated [66]. Similarly, characterization of the bovine serum albumin (BSA)-derived peptide FW and its related sequence FWGK, which were effectively produced by tryptic digestion of BSA, demonstrated the higher antihypertensive effect of the tetrapeptide in comparison to that of FW [67].

Although the in vivo efficacy of antihypertensive peptides is usually hampered by low bioavailability, few attempts to identify descriptors of intestinal stability and permeability of ACE inhibitory peptides have been conducted. In this context, QSAR modeling of dipeptides identified the amino acid residues G, P and D at the N-terminus as determinants of intestinal stability, independently of the amino acid located on the C-terminus [68]. However, QSAR modeling failed to identify predictors of intestinal permeability due to the very low and similar transport properties of the different peptides tested [68].

### 2.2. Antidiabetic Peptides

There are reports on the inhibition of DPP-IV by different food-protein-derived peptides and their potential in the management of type 2 diabetes, although the number of peptide sequences is still limited and few studies link in vitro enzyme inhibition and in vivo antidiabetic effects [15,16]. However, from the attempts to elucidate the structure–activity relationships of DPP-IV inhibitors and the parallelism with the approaches taken for ACE inhibitory peptides, some important conclusions are reached (Figure 3b). DPP-IV is a proline/alanine-specific peptidase cleaving after XP and XA (where X represents any amino acid) at the N-terminus of peptides [69], and dipeptides that mimic the DPP-IV substrate are potential inhibitors. Hikida and coworkers [70] carried out a systematic analysis of XP and XA dipeptides. This approach allowed the selection of WP and WA among sequences exhibiting the highest inhibitory effect on DPP-IV activity, and a second screening performed for WX dipeptides yielded WP and WR as the most effective inhibitory peptides [70]. Later on, and by comparison of WR with a complete set of WRX tripeptides, it was established that the inhibitory effect of the dipeptide was higher than that of tripeptides [71]. Although less active than dipeptides, all of the tripeptides evaluated showed unique uncompetitive-type inhibition in contrast to the competitive-type inhibition showed by WP and the mixed-type inhibition provoked by WR, suggesting these tripeptides as lead sequences for DPP-IV inhibitors with a mechanism of action different to that of dipeptides. Among tripeptides, the sequence WRE showed the highest inhibition of DPP-IV. Its presence in the primary sequence of soybean β-amylase suggests its release from soy protein following enzymatic digestion [71]. Given the higher inhibition of dipeptides over tripeptides, an exhaustive analysis of a complete dipeptide library was conducted and confirmed that amino acid residues at the N-terminus determine the inhibitory potency [72]. In addition to the previously W-containing dipeptides, this approach allowed the selection of novel sequences such as TH, NH, VL, ML, and MM [71]. Interestingly, the relevance of aromatic amino acids with polar side-chain and P at the N-terminus (such as WP but also YP) was also deduced from sequence alignment of different DPP-IV inhibitory peptides from food proteins [73,74]. All these sequences were screened in dietary proteins to select canola, chicken egg, oat, wheat [73], soy [72] and β-lactoglobulin [74] as potential sources of DPP-IV inhibitory peptides. Later on, these structural features and a QSAR approach were used to predict the DPP-IV inhibitory potential of milk protein-derived peptides identified in the intestine of humans [75]. Although the approach did not allow an accurate prediction of the DPP-IV inhibitory potency, peptides possessing a hydrophobic N-terminal amino acid (W, I, F and L) were predicted to be relatively potent inhibitors of DPP-IV, in agreement with previous studies [70,73,74].

Due to the still low number of available sequences of α-glucosidase-inhibitory peptides, it is not easy to determine the structural requirements for enzyme inhibition. Based on the sequence and potency of already described food-derived α-glucosidase-inhibitory peptides, the requirements for optimal enzyme inhibition were hypothesized. Requirements included a sequence of three to six amino acid residues with either S, T, Y, K, or R at the N-terminus and a P residue closer to the C-terminal with residues M or A occupying the ultimate C-terminal position [76]. Therefore, these previously identified structural properties were used to design a library of 4,210 peptides containing 3–5 amino acid residues, with potential α-glucosidase inhibitory activity [77]. From all the designed peptides, only those resistant to in silico gastrointestinal digestion were subjected to molecular docking analysis. Finally, two tetrapeptides (SVPA and SEPA) that had the highest binding affinity were selected. Interestingly both peptides were also α-amylase inhibitors [77].

Multifunctional antidiabetic sequences were also identified through a systematic evaluation of peptides derived from black bean proteins by computational docking analysis [78]. Sequences AKSPLF, QTPF, FEELN and LSKSVL were selected based on their potential inhibitory activities of DPP-IV, α-amylase and α-glucosidase through hydrogen bonds, polar and hydrophobic interactions. Table 2 summarizes predicted and novel antidiabetic sequences obtained by computational methods and rational design.

### 2.3. Antioxidant Peptides

Since the antioxidant effect of peptides was reported [79], hundreds of antioxidant peptides generated from the digestion of dietary proteins have been identified. Several amino acids such as Y, M, H, K, and W, are generally accepted to be antioxidant and exhibit higher activities when incorporated into peptides, but neither the structure activity relationship nor the mechanism of action of antioxidant peptides is fully understood. Moreover, the lack of a standardized methodology to evaluate in vitro antioxidant activity hampers the comparison of results from different laboratories [20,80,81].

Several attempts to explore the sequence-activity relationship of antioxidant peptides have been reported (Figure 3c and Table 3). Based on the soybean protein-derived antioxidant pentapeptide LLPHH, 28 structurally related peptides were synthesized and their activities against the peroxidation of linoleic acid in an aqueous system were compared. The deletion of the C-terminal H residue decreased the activity while the deletion of the N-terminal L did not have any effect [82]. This approach established the relevant role of P and H residues in the antioxidant activity, and the fragment PHH was identified as the active core of the antioxidant peptide [82]. Although the amino acid Y is known to be an antioxidant by itself, sequence variants obtained by P or H replacement with Y (sequences YHH and PYY) unexpectedly lost part of the antioxidant effect. Other Y-containing peptides, such as LPYY, LYPY, and YLYP, were also less active than the corresponding H-containing sequences LPHH, LHPH and HLHP [82]. However, when the antioxidant activity of the previously selected PHH sequence was examined in other oxidation systems, the peptide did not show activity [83]. This finding pointed to the need of using different assays for determination of antioxidant activity as it was done in an additional study in which two series of combinatorial tripeptide libraries were constructed [84]. One library was composed by 114 peptides structurally related to PHH and the other was composed of 108 peptides containing either two H or Y residues in the sequences. Both libraries were screened by different methods, including the antioxidant activity against the peroxidation of linoleic acid, the reducing activity, the radical scavenging activity, and the peroxynitrite scavenging activity. The study showed that tripeptides had distinct activities in different assay systems, but several unique sequences such as YHY, XXW, XXY and XXC could be identified that had synergistic effect with phenolic antioxidants, high radical scavenging activity and high peroxynitrite scavenging activity, respectively [84].

The relevance of H and Y residues on antioxidant activity was also confirmed by designing the peptides ECH and YECG based on the sequence of the natural nonribosomal tripeptide glutathione (ECG). The tripeptide ECH displayed the highest DPPH radical scavenging activity, the strongest reducing power and the best inhibition activity toward linoleic acid peroxidation, whereas the tetrapeptide YECG showed the highest oxygen radical absorption capacity and ABTS free radical scavenging ability and the best protection in cell cultures [86]. The presence of Y, W, C or M residues in antioxidant dipeptides was the driving force for the radical scavenging activity [87], whereas a tripeptide QSAR study confirmed W, Y or C at the N-terminal position as favorable residues for antioxidant capability evaluated in three antioxidant assays [85]. Moreover, designed Y- and W-containing antioxidant peptides (YGY, YGGY, GYYG, GWWW) based on the antioxidant activities of free amino acids supported the relevance of both residues [88]. The positive role of Q residues as well as that of QP and PY pairs was inferred from a structure-function relationship study based on the antioxidant barley-derived pentapeptide QPYPQ [18]. This study revealed not only the importance of the chemical structure of individual side chains but also the combined effects of the vicinal residues in the antioxidant activity of peptides. Besides, the structure–activity relationship of the sesame protein-derived peptide SYPTECRMR established the fragment ECRMR as the antioxidant active sequence and the relevance of C and M residues on the activity. The study also attributed the antioxidant activity of SYPTECRMR to the C-terminal R residue [89]. Remarkably, all these studies highlighted the necessity of examining the antioxidant activity of peptides in different assay systems for a comprehensive evaluation of sequence-activity relationships and the antioxidant mechanisms of peptides.

## 3. Oral Delivery Strategies

Bioavailability of orally ingested nutraceuticals may affect their bioactive impact [90]. The health-promoting effects of bioactive peptides depend on the ability to cross the intestinal mucosa and reach their target organs in an active form. This means resistance to gastrointestinal enzymes and brush border membrane peptidases, absorption through the intestinal barrier associated to the resistance to intracellular peptidases and stability into the systemic circulation. In addition, as components of functional foods, bioavailability of peptides can be affected by diet. Pharmaceutical approaches to circumvent these challenges (Figure 2) have resulted in the development of several new protein- and peptide-based therapeutics [91], and the application of these approaches such as permeation enhancers and delivery systems to food-derived bioactive peptides is a field of interest [92,93,94]. Relevant examples of delivery strategies to improve bioavailability of food-derived bioactive peptides are summarized below and in Table 4.

Regarding permeation enhancers, sodium caprate (C_10_), the gold standard in clinical trials, increased in vitro permeability of the food-derived antihypertensive peptides VPP and LKP across rat intestinal epithelium [95]. Authors reported that C_10_ increased the permeation of fluorescein isothiocyanate (FITC)-labelled IPP and LKP by 1.4–3.6-fold, respectively, across isolated rat jejunal tissue mucosae. Moreover, authors demonstrated that C_10_ overcomes the decrease in the intestinal permeability of IPP and LKP provoked by the presence of a complex mixture of peptides in food, which act by inhibiting PepT1, the intestinal transporter for di- and tripeptides [96]. In order to mimic the inhibition of PepT1 due to protein hydrolysates, intestinal transport of IPP and LKP was evaluated in the presence of the PepT1 inhibitor glycyl-sarcosine (Gly-Sar) [97]. After oral administration to normotensive rats, plasma levels of both peptides were reduced when co-administered with Gly-Sar but restored in the presence of C_10_. Moreover, similar results were obtained in SHRs where the antihypertensive effect provoked by IPP and LKP was diminished by the inhibitor and re-established by C_10_ through the enhancement of paracellular permeability. These studies are a smart demonstration that a food-grade agent as C_10_, naturally present in milk and oils, can enhance in vivo effects of food-derived bioactive peptides and paves the way for the design of optimized oral formulations and/or functional foods containing both nutraceuticals and intestinal permeation enhancers.

Food-derived bioactive peptides may also benefit from delivery systems such as encapsulation, which has been shown to be an effective method to mask the unfavorable taste as well as improve the solubility and stability of functional ingredients via the oral route [98,99]. Encapsulation in nanoparticles has been described for individual peptides and complex mixtures (Table 4). Poly-(lactic-co-glycolic) acid (PLGA) nanoparticles, liposomes and combination of both in lipid nanoparticles have attracted much attention, due to biodegradability, biocompatibility, nontoxicity and sustained release properties.

Encapsulation of individual peptides includes those with antihypertensive effect (Table 4). For instance, the antihypertensive pentapeptide VLPVP was encapsulated in a PLGA-based nanoparticle, and exhibited sustained attenuation of hypertension after oral administration to SHRs compared to free peptide [100]. A similar strategy was followed with the antihypertensive dipeptide FY previously isolated from a peptic digest of wakame [115]. Although encapsulation of FY in PLGA nanoparticles improved peptide toxicity profile in fibroblast cells, no data about bioavailability or antihypertensive effect enhancement were provided [101]. Encapsulation in liposomes for the improvement of intestinal bioavailability was shown to be successful for the ACE inhibitory milk-derived hexapeptide RLSFNP [102]. The apparent permeability value (Papp) of RLSFNP liposomes was higher than that of the free peptide showing that a liposome carrier can improve transport into the Caco-2 monolayer. Also successful was the entrapment into lipid nanoparticles of the milk protein-derived antihypertensive tetrapeptide YGLF. Optimal nanoliposomes exhibited a sustained in vitro release of the peptide and, most importantly, the nanoparticles provoked a five-day, long-term antihypertensive effect in SHRs [107].

Much more information is available for encapsulation of complex mixtures with mainly antihypertensive, but also antidiabetic, antioxidant or immunomodulatory activities (Table 4). The enhancing effects of different approaches to improve the intestinal absorption of oligopeptides from tuna cooking juice were evaluated in vivo by oral administration in SHRs. Although the different approaches were feasible, the enhancing effect provoked by encapsulation in liposomes was more effective than those of surfactants and mucoadhesive substances. Liposomes at a half-dose of that of the non-encapsulated oligopeptides exerted a higher reduction in SBP and long-lasting effect in SHRs [103]. Later on, and with the aim of reducing cholesterol concentration in liposomes, the suitability of phytosterol instead of cholesterol for encapsulating tuna cooking juice oligopeptides was evaluated. Encapsulated oligopeptides in phytosterol-containing liposomes orally administered to SHRs provoked a sustained blood-pressure-lowering effect, similar to that caused by cholesterol liposomes [104].

Stone fish-derived ACE inhibitory peptides were stabilized by encapsulation in sodium tripolyphosphate (TPP) cross-linked chitosan nanoparticles. These nanoparticles showed a sustained in vitro release and enhanced physicochemical stability as well as improved inhibitory effect against ACE following simulated gastrointestinal digestion [110]. Besides, results of the in vivo efficacy of these chitosan nanoparticles in SHRs indicated a dose-dependent blood pressure lowering effect that was significantly higher than that caused by the non-encapsulated peptides [111]. In addition, the encapsulation of stone fish-derived bioactive peptides in nanoliposomes improved peptide stability against in vitro gastrointestinal digestion, although no data about the antihypertensive effect were provided [112]. Similarly, improved stability and bioavailability of nanoliposomes loaded with an ACE inhibitory peanut peptide fraction in a simulated gastrointestinal tract were reported [108]. Also for antidiabetic hydrolysates, chitosan coated-liposomes were effective in extending the release of peptides in simulated biological fluids, although the retention of the bioactivity of the encapsulated peptides was not tested [105].

Encapsulation structures for the protection of bioactive hydrolysates during food processing were assessed. In this context, a whey protein hydrolysate reported to exert different bioactivities was used as a model to study the impact of microencapsulation on protection during in vitro digestion but also during milk fermentation [113]. Although peptide bioaccessibility was not compromised by encapsulation within microparticles, no protective effect during in vitro digestion was demonstrated. Moreover, encapsulation provided a partial protection during milk lactic acid fermentation that was sequence- and matrix-dependent, suggesting that the encapsulation matrix should be selected based on the specific bioactive sequence to be protected [113]. By contrast, spray-drying of an immunomodulatory hydrolysate derived from whey protein concentrate maintained the bioactivity in in vitro assays, while reducing the bitter taste and hygroscopicity, suggesting the feasibility of the microencapsulated hydrolysate to be used as a food ingredient [114].

Another interesting strategy is the incorporation of liposomes containing bioactive peptides in gelatin, which is considered as a highly digestible dietary food [109]. In particular, phosphatidylcholine nanoliposomes containing ACE inhibitory peptides were incorporated in fish gelatin, conferring the bioactivity while preserving the rheological properties and thermal stability of this nanoliposome-containing fish gelatin. In addition, the use of mucoadhesive edible films carrying bioactive peptides may represent a smart approach for buccal delivery. In this sense, soy phosphatidylcholine liposomes containing a low molecular weight peptide fraction with antioxidant and ACE- and DPP-IV inhibitory activities were incorporated in an edible film of sodium caseinate [106]. The resulting films had appropriate sensory characteristics and enhanced palatability, although the stability of the encapsulated bioactive peptides was not discussed.

## 4. Conclusions and Perspectives

Data reported here demonstrate the potential application of common pharmaceutical approaches such as rational design and oral delivery strategies for improving the health-promoting effects of food-derived bioactive peptides (Figure 2). Rational design has been successfully used in drug discovery, but it is still challenging for food-derived bioactive peptides. One of the major challenges is that the mechanisms underlying the effects of bioactive peptides are not fully established and seem not only limited to one molecular target (Figure 1). Besides, integrated computational methods are mainly focused on one target. In addition, most of the relevant predicted/novel sequences discussed here have been evaluated in in vitro assays, and data about in vivo effects are still lacking. Undoubtedly, further research will link in silico and in vitro studies with the efficacy of these peptides in animal and human clinical studies.

Despite these challenges, structural requirements for bioactive peptides reviewed here highlight the relevance of specific amino acid residues such as W, P, F, L and Y within the sequence of antihypertensive, antidiabetic and/or antioxidant peptides (Figure 3). Data also demonstrate that changes in even a single amino acid could alter the biological function completely, suggesting that each peptide might have a unique mode of action determined by the peptide structure. Particularly, integrated computational methods underscore the important role of W for the bioactivity. Moreover, W-containing peptides as those described here display other biological activities including antimicrobial, neuroprotective and satiating properties [116,117,118], suggesting the multifunctionality of such peptides which might function as polypharmacological sequences. Whether novel natural and rationally designed W-containing peptides are suitable starting leads to design effective agents for preventing or postponing lifestyle-associated diseases deserves future research. Sequence-activity studies also allow the identification of the most appropriate food protein sources for the production of bioactive peptides. In this sense, the presence of some of the novel bioactive peptides within the primary sequence of dietary proteins opens the way for targeted generation of bioactive peptides during hydrolysis of food proteins and for the future inclusion of such proteins/hydrolysates in dietary recommendations.

Peptide delivery and gastrointestinal absorption remain crucial for application of food-derived bioactive peptides. This review gives relevant examples of effective oral delivery systems to enhance solubility, stability, and permeability providing a useful platform for facilitating the use of food-derived bioactive peptides in the industry. However, there is no a universal delivery system for all bioactive peptides, since the final efficacy is dependent on the sequence, the system and the food matrix. In addition, to maximize the potential of food-derived peptide delivery strategies, pharmacokinetic studies and more in vivo evidence of efficacy are mandatory and still lacking in many of the studies summarized above. Still challenging is the safety evaluation and optimization of delivery strategies. In conclusion, progress has been made in the field of food-derived bioactive peptides applying pharmaceutical technologies widely used in drug discovery. It is expected that in the near future, all of this scientific knowledge will facilitate the use of bioactive peptides to improve human health and wellness.

## Figures and Tables

**Table 1:** Predicted/novel antihypertensive sequences obtained by integrated computational methods and rational design.

Table	Predicted/Novel Sequence ^1^	Methodology	Evaluation	Ref.
ACE inhibition	**FW, WW, YW, VRF, IKP, LRW, LRF**	QSAR modeling	In vitro IC_50_	[35]
	CW, TW, HW, QW, CY	QSAR modeling	In vitro IC_50_	[36]
	**DW, WP**	Molecular docking	In vitro IC_50_	[37]
	**KW, VW, MKP, FAP, VAP,**	Molecular docking	In silico IC_50_	[38]
	**IPP, VPP, LRP, IVY**			
	WCW, IWW, WWW, WWI, WLW	Tripeptide library, molecular docking	In vitro IC_50_	[39]
	VKW, YAW, KYW, TAW	Rational design	In vitro IC_50,_ cell toxicity	[40]
	**IVP, INP, IQP, VIP**	QSAR modeling	In vitro IC_50,_ SHRs	[41]
	VPPIPP, IPPVPP	Rational design	In vitro IC_50,_ SHRs	[42]
	GEF, VEF, VRF, VKF	QSAR modeling, molecular docking	In vitro IC_50_	[43]
	RKWHFW, RKWLFW	Partial hexapeptide library	In vitro IC_50_, vasoconstriction, SHRs	[44]
	RKWHFLW	Rational design	In vitro IC_50_, vasoconstriction,	[45]
			SHRs, toxicity	
	LHLPGP, LHLPLR	Rational design	In vitro IC_50_	[46]
Renin inhibition	IW, LW, VW, AW	QSAR modeling	In vitro renin activity	[47]
ACE & renin	RYLP, YTAWVP, YRAWVL	QSAR modeling, molecular dynamics,	In vitro IC_50_	[48]
inhibition		peptide binding free energy		

^1^ In bold those that are found within the primary sequence of food proteins.

**Table 2:** Predicted/novel antidiabetic sequences obtained by integrated computational methods and rational design.

Table	Sequence ^1^	Methodology	Evaluation	Ref.
DPP-IV inhibition	**WP**, WA, WR	XP and XA library	In vitro inhibitory effect	[70]
	**WRE**	WRX library	In vitro inhibitory effect	[71]
	**TH, NH, VL,**	Dipeptide library	In vitro inhibitory effect	[72]
	**ML, MM**			
	**WP, YP**	Sequence alignment	In vitro inhibitory effect	[73]
α-glucosidase	**SVPA, SEPA**	Tri-tetra- and	In vitro IC_50_	[77]
inhibition		pentapeptide library		
DPP-IV, α-glucosidase,	**AKSPLF, QTPF,**	Computational docking	In vitro inhibitory effect	[78]
α-amylase imhibition	**FEELN, LSKSVL**	analysis		

^1^ In bold those that are found within the primary sequence of food proteins.

**Table 3:** Predicted/novel antioxidant sequences obtained by integrated computational methods and rational design.

Sequence ^1^	Methodology	Evaluation	Ref.
**PHH**	LLPHH-related peptides	Activity against peroxidation of linoleic acid	[82]
YHY, XXW,	Tripeptide library,	Activity against peroxidation of linoleic acid,	[84,85]
XXY, XXC	QSAR modeling	reducing activity, radical and peroxynitrite	
		scavenging activity, Trolox equivalent	
		antioxidant capacity (TEAC), ferric	
		reducing antioxidant activity	
ECH, YECG	Rational design	Radical scavenging activity, reducing power,	[86]
		activity against peroxidation of linoleic acid,	
		oxygen radical absorbance capacity (ORAC),	
		TEAC, protection on H_2_O_2_-induced cytotoxicity	
YX, XY,	Library of Y-, W-, C-	Radical scavenging activities, reducing power,	[87]
WX, XW	or M-containing dipeptides,	iron chelating activity, protective effect on	
	QSAR modeling	erythrocyte hemolysis	
YGY, YGGY,	Rational design	Antioxidant activities against hypochlorite ion,	[88]
GYYG, GWWW		hydroxyl radical, peroxynitrite	
**QP, PY**	Rational design	Radical scavenging activity, iron chelating	[18]
		activity, ORAC, cell response studies	
**ECRMR**	Rational design,	Radical scavenging activity	[89]
	3D-QSAR modeling		

^1^ In bold those that are found within the primary sequence of food proteins.

**Table 4:** Oral delivery strategies applied to food-derived bioactive peptides.

Delivery Strategy	Peptide (Origin)/Hydrolysate	Bioactivity	Evaluated Functionality	In vitro/In vivo Model	Ref.
Sodium caprate	VPP (milk) and LKP (chicken, fish)	Antihypertensive	Intestinal permeability, antihypertensive effect	Rat jejunal tissue, plasma levels, SHRs	[95,96,97]
PLGA-based nanoparticles	VLPVP (synthetic)	Antihypertensive	Antihypertensive effect	SHRs	[100]
	FY (seaweed)	Antihypertensive	Peptide toxicity	Fibroblast cells	[101]
Liposomes	RLSFNP (milk)	ACE-inhibitory	Intestinal transport	Caco-2 cells	[102]
	Tuna cooking juice oligopeptides	Antihypertensive	Antihypertensive effect	SHRs	[103,104]
Chitosan coated liposomes	Salmon protein hydrolysate	Antidiabetic	In vitro release	Simulated biological fluids	[105]
Liposomes in sodium caseinate films	Shrimp peptide fraction	Antioxidant, ACE- and DPP-IV inhibitory	Solubility, palatability	Sensory evaluation	[106]
Nanoliposomes	YGLF (milk)	Antihypertensive	In vitro release, antihypertensive effect	SHRs	[107]
	Peanut peptide fraction	ACE inhibitory	In vitro release, stability, bioavailability	Gastrointestinal digestion	[108]
Nanoliposomes in fish gelatin	Squid tunic hydrolysate	ACE inhibitory	Stability, ACE inhibition	In vitro ACE inhibition	[109]
Nanoliposomes & chitosan nanoparticles	Stone fish-derived peptides	ACE inhibitory	In vitro release, stability, ACE inhibition, antihypertensive effect	Gastrointestinal digestion, SHRs	[110,111,112]
Microencapsulation in gelatin and chitosan	Whey protein hydrolysate	ACE-, DPP-IV inhibitory, hypocholesterolemic, antimicrobial	Bioaccesibility, stability	Gastrointestinal digestion, fermentation	[113]
Microencapsulation in sodium alginate and whey protein concentrate	Whey protein hydrolysate	Immunomodulatory	Immunomodulation, bitterness, hygroscopicity	*In vitro* splenocyte proliferation	[114]
